# Marmal-aid – a database for Infinium HumanMethylation450

**DOI:** 10.1186/1471-2105-14-359

**Published:** 2013-12-12

**Authors:** Robert Lowe, Vardhman K Rakyan

**Affiliations:** 1 The Blizard Institute, Barts and The London School of Medicine and Dentistry, Queen Mary University of London, London, UK

## Abstract

**Background:**

DNA methylation is indispensible for normal human genome function. Currently there is an increasingly large number of DNA methylomic data being released in the public domain allowing for an opportunity to investigate the relationships between the DNA methylome, genome function, and human phenotypes. The Illumina450K is one of the most popular platforms for assessing DNA methylation with over 10,000 samples available in the public domain. However, accessing all this data requires downloading each individual experiment and due to inconsistent annotation, accessing the right data can be a challenge.

**Description:**

Here we introduce ‘Marmal-aid’, the first standardised database for DNA methylation (freely available at http://marmal-aid.org). In Marmal-aid, the majority of publicly available Illumina HumanMethylation450 data is incorporated into a single repository allowing for re-processing of data including normalisation and imputation of missing values. The database is accessible in two ways: (1) Using an R package to allow for incorporation into existing analysis pipelines which can then be easily queried to gain insight into the functionality of certain CpG sites. This is aimed at a bioinformatician with experience in R. (2) Using a graphical interface allowing general biologists to query a pre-defined set of tissues (currently 15) providing a reference database of the methylation state in these tissues for the 450,000 CpG sites profiled by the Illumina HumanMethylation450.

**Conclusion:**

Marmal-aid is the largest publicly available Illumina HumanMethylation450 methylation database combining Illumina HumanMethylation450 data from a number of sources into a single location with a single common annotation format. This allows for automated extraction using the R package and inclusion into existing analysis pipelines. Marmal-aid also provides a easy to use GUI to visualise methylation data in user defined genomic regions for various reference tissues.

## Background

DNA methylation particularly in CpG Islands located near promoters has long been known to repress gene expression [1], however, the role of methylation outside these regions is still not well understood. It is essential for the development of mamals [2] and its importance is further demonstrated by recent epigenome-wide association studies (EWAS) that have identified DNA methylation signatures for different human pathologies [3]. As a consequence, there is now a vast amount of DNA methylomic data in the public domain, providing unprecedented opportunities to use computational approaches to uncover novel and fundamental relationships between the DNA methylome, genome function, and human phenotypes. However, till now, extracting this data for analysis in specific pipelines has required downloading each individual experiment by hand with limited ability to search due to inconsistent annotation.

**Figure 1:**
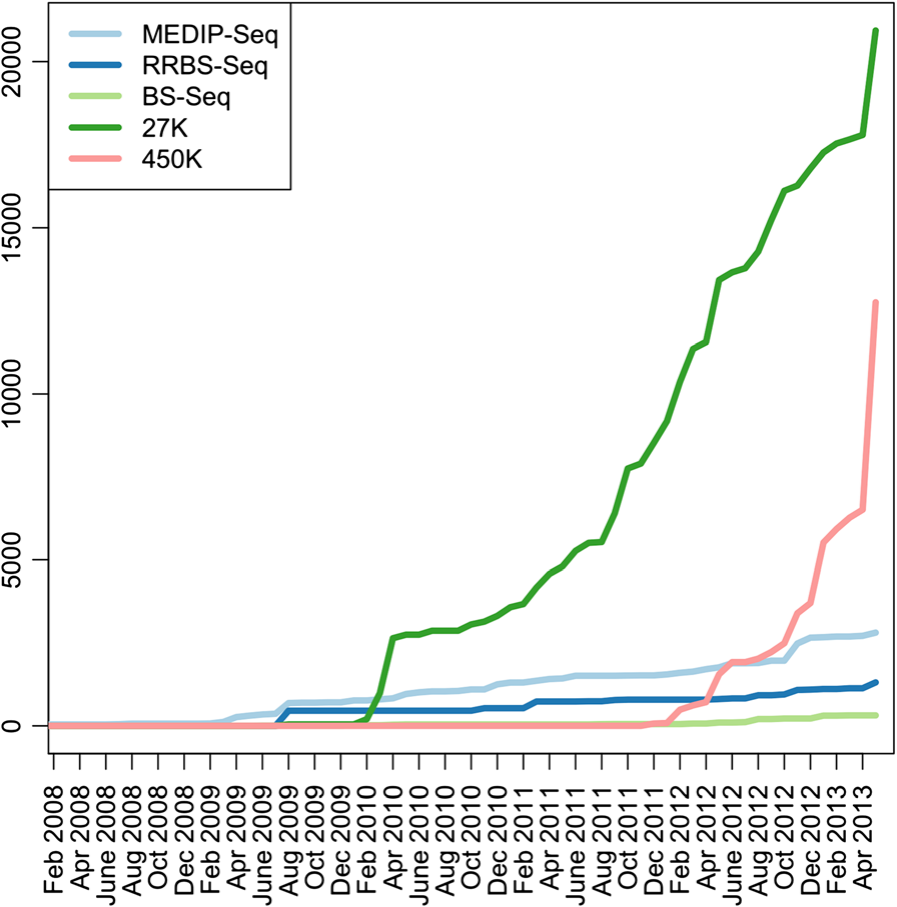
**The growth of methylation data in the public domain for different assays.** The number of samples contained within GEO was extracted for Illumina 27K Array (GPL8490) and Illumina HumanMethylation450 array (GPL13534). “MEDIP”, “BS-Seq” and “RRBS” were used as search terms with a filter for Homo sapiens to extract the number of samples for MEDIP-Seq, RRBS-Seq and BS-Seq. The cumulative number of samples for each month from February 2008 to June 2013 is shown for all of the different assays.

Of the various DNA methylomic technologies that have been developed in the last few years [4], the Illumina Infinium platform is the most popular **(**Figure 1**)**[5]. The Illumina450K array permits genome-scale interrogation of over 450,000 CpG sites associated with nearly all known human promoters, genes, CpG islands, and non-genic features including enhancers. As of June 2013, data from >10,000 different arrays are in the public domain, and this number is rising exponentially. The diversity of data spans small-scale projects that focus on, for example, Progeria, iPS, cellular senescence, twin discordance, to large-scale EWASs and comprehensive mapping efforts (The Cancer Genome Atlas and ENCODE).

Here we report the first dedicated DNA methylomic database called Marmal-aid. In Marmal-aid, all publicly available Illumina450K data is incorporated into a single repository and re-processed. This enables a wide variety of customizable meta-analyses to gain insights into the functionality of key CpG sites, without having to rely on gene-centric approaches such as Gene Ontology (GO). Marmal-aid has the potential to become a powerful database that will work both independently and in combination with GO and/or gene pathway analyses in the context of a variety of epigenomic investigations such as EWAS, genetic-epigenetic interactions, and environmental epigenomics.

## Construction and content

We first extracted all Illumina HumanMethylation450 data from Gene Expression Omnibus (GEO). The most common format available for these arrays is a reported beta value. The beta value is a commonly used measure of methylation ranging from 0-1 and is calculated using eq 1.

$$\beta =\frac{I_{M}}{I_{M}+I_{U}+100}$$

**Figure 2:**
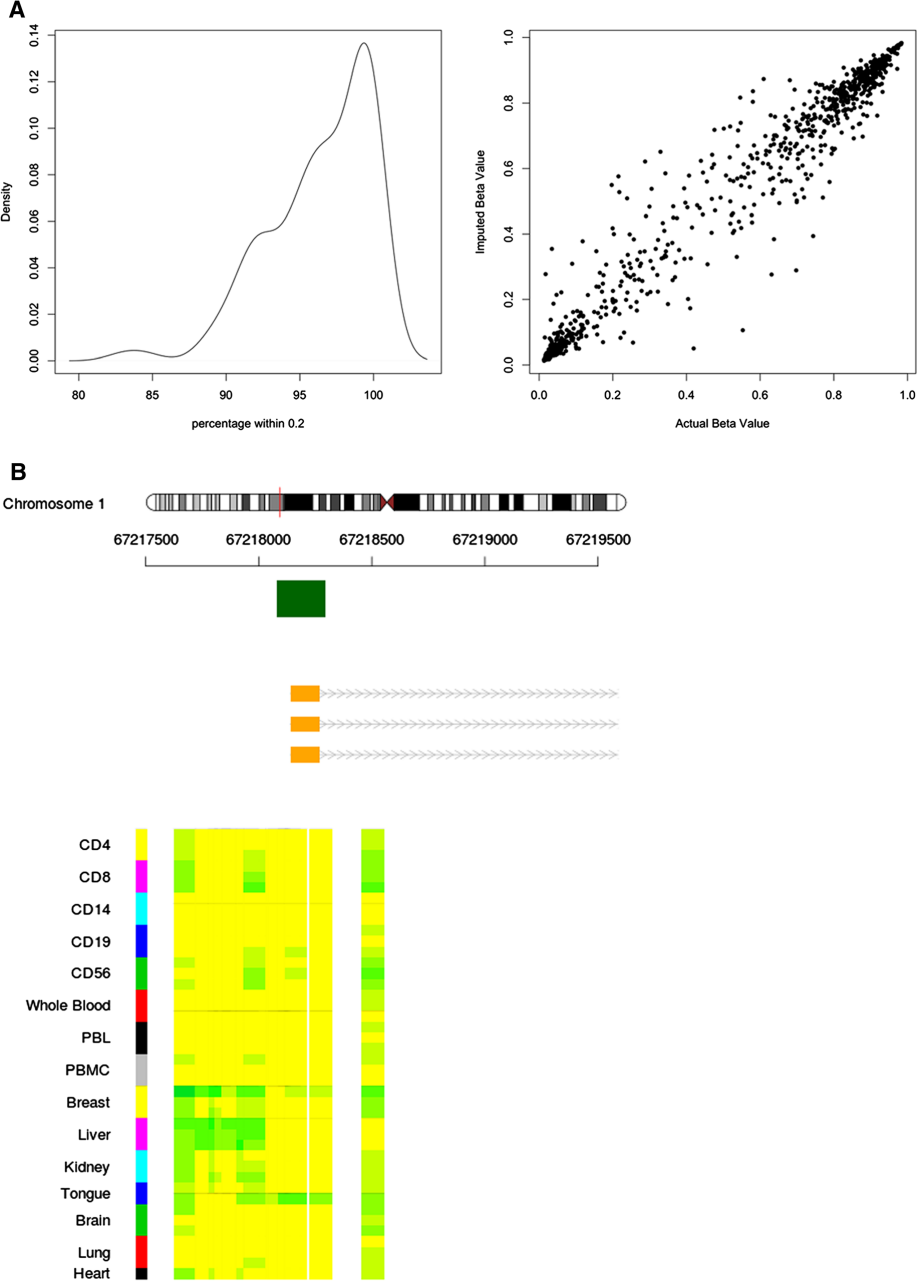
**Imputation and visualisation in Marmal-aid. (A)** The left panel shows the percentage of probes that were imputed to within 0.2 of the actual beta value for over 100 different random samples. On average over 96% of the probes were imputed to within 0.2 of the actual value. The right panel shows an example of 1 of the 100 random samples in which the actual beta value is plotted on the x-axis and the imputed value on the y-axis. **(B)** An example of the GUI available at http://marmal-aid.org/visualise.html for chr1:67,217,505-67,219,505 around the TSS of TCTEX101.

Where *I*_*M*_, *I*_*U*_ are the intensities of the methylated and unmethylated channels respectively. We further supplemented this data with arrays extracted from The Cancer Genome Atlas (TCGA, http://cancergenome.nih.gov/) that provide intensity files. These were transformed into beta values using eq 1. We removed arrays which contained values from GEO outside of the 0-1 range which are presumably M-Values and arrays from TCGA which had no known annotation. To annotate the data we created a single table with annotations such as tissue, tissue subtype, sex, age and disease status as well as a column with additional annotation (Table 1), all manually verified with the original publication. Arrays were annotated by extracting the appropriate information from GEO and manually converting this annotation to be inline with our table. While all the arrays are available from Marmal-aid as beta values, as extracted from GEO and TCGA, we also apply our own normalisation as an attempt to decrease variability across experiments. We used a standard quantile normalisation procedure but due to the large number and variety of samples, which may have significantly different beta distributions, we normalised each array using only a subset of the data. To do this, we first calculated the Euclidean distance between each of the samples on a random sampling of 10,000 probes. Clustering on the full dataset requires very large amounts of RAM or CPU time and hence we repeated this clustering a number of times and found the samples were consistently clustered together in the same groups. We then calculated for each sample the 25 nearest neighbours. We then used these 25 neighbouring arrays to impute missing data (see below) and then to perform quantile normalisation. A small number of probes will not register a reading above background and these probes are generally reported as missing data in the public repositories. While this is not a problem for a small study, when investigating thousands of samples it is more likely that at least a single sample will contain a missing value in the majority of probes and hence we used a k-nearest neighbour technique to impute the data [6]. To test the imputation we randomly chose a sample and extracted the raw beta values that contained no missing values. We then randomly removed 1,000 probes and used the method as described above (without quantile normalisation) to impute the artificially missing values using the 25 nearest neighbours. This was repeated 100 times and we found that 97% of the imputed values were within 20% of the original (Figure 2A).

**Table 1:** A description of the column names contained in the annotation file in Marmal-aid

**Column name**	**Description**
**ID**	ID used to identify sample. If from GEO this is the GEO accession number
**SAMPLE_NAME**	A long descriptive name of the sample
**GSE**	The GSE number which can be used to a certain whether samples are from the same experiment.
**LINEAGE**	The lineage of the tissue
**TISSUE**	Main tissue type E g. Blood, Brain, Liver, Kidney
**TISSUE_SUBTYPE**	Further sub categorisation of the tissue E g. for Blood this may be CD19, CD4 etc
**TRANSFORMED**	If a transformed cell line the name of the line is given in this column
**DISEASE**	Indication of the disease state of the sample. NA here means no information was recorded and is most likely healthy.
**DISEASE_SUBTYPE**	A further charaterisation of the disease state E g. if the DISEASE column contains Cancer then this column will indicate what type
**SEX**	The sex of the sample if given as taken from the annotation file
**SEX_PRED**	The prediction of the sex of the sample using autosomal probes
**AGE**	The age of the sample if given.
**ADDITIONAL**	Any additional information that could not be described in the other columns.

## Utility and discussion

The website http://marmal-aid.org provides the location for the R package as well as tutorials, important update information, samples page with a real time searchable table and a forum. A particularly useful part of the website is the methylation reference visualisation which provides a GUI interface to examine the methylation of a reference set of samples. This GUI was designed to allow a biologist with limited knowledge of bioinformatics to view the methylation state of a number of reference tissues at a particular gene or genomic location. A user can supply genomic co-ordinates of interest and the methylation state of probes contained on the array within these co-ordinates will be shown. This tool can be used to quickly ascertain whether a particular gene is differentially methylated in different tissues. For example at chr1:67,217,505-67,219,505 around the TSS of TCTEX101 we find an unmethylated CpG Island in the majority of tissues (Figure 2B).

For more in-depth analysis the R package allows an easy interface to access samples and quickly analyse them in existing pipelines. The R package can be installed using a single command [install.packages(“directory_of_download/marmalaid_1.2.tar.gz”,repos = NULL,type = “source”)]. To extract data from Marmal-aid then requires a single command, [beta = getbeta(samples,probes)], where samples are a vector of ids obtainable from the annotation file e g. (“GSM1052413”, “GSM1052414”,“GSM1052415”) and probes are the Illumina IDs of the probes required e g. “cg09432775” or ”cg03134653”. A more detailed tutorial is provided on the website. We currently provide a small number of functions that we have found useful such as a heatmap visualisation of the methylation state of each of your samples and probes. Also while Marmal-aid is useful for investigating the methylation state of a large number of samples in comparison to a 450K study design it may also be used to quickly assaying the methylation state of a region of interest (perhaps from an RNA-Seq experiment) and hence it is possible to input genomic co-ordinates instead of probes.

By allowing easy programmable access to Marmal-aid and hand curated annotation data we envision it has the potential to become a powerful database to be used as a meta-analytical tool that could work both independently and in combination with GO and/or pathway analyses in the context of a variety of epigenomic investigations such as EWAS, genetic-epigenetic interactions, and environmental epigenomics.

**Figure 3:**
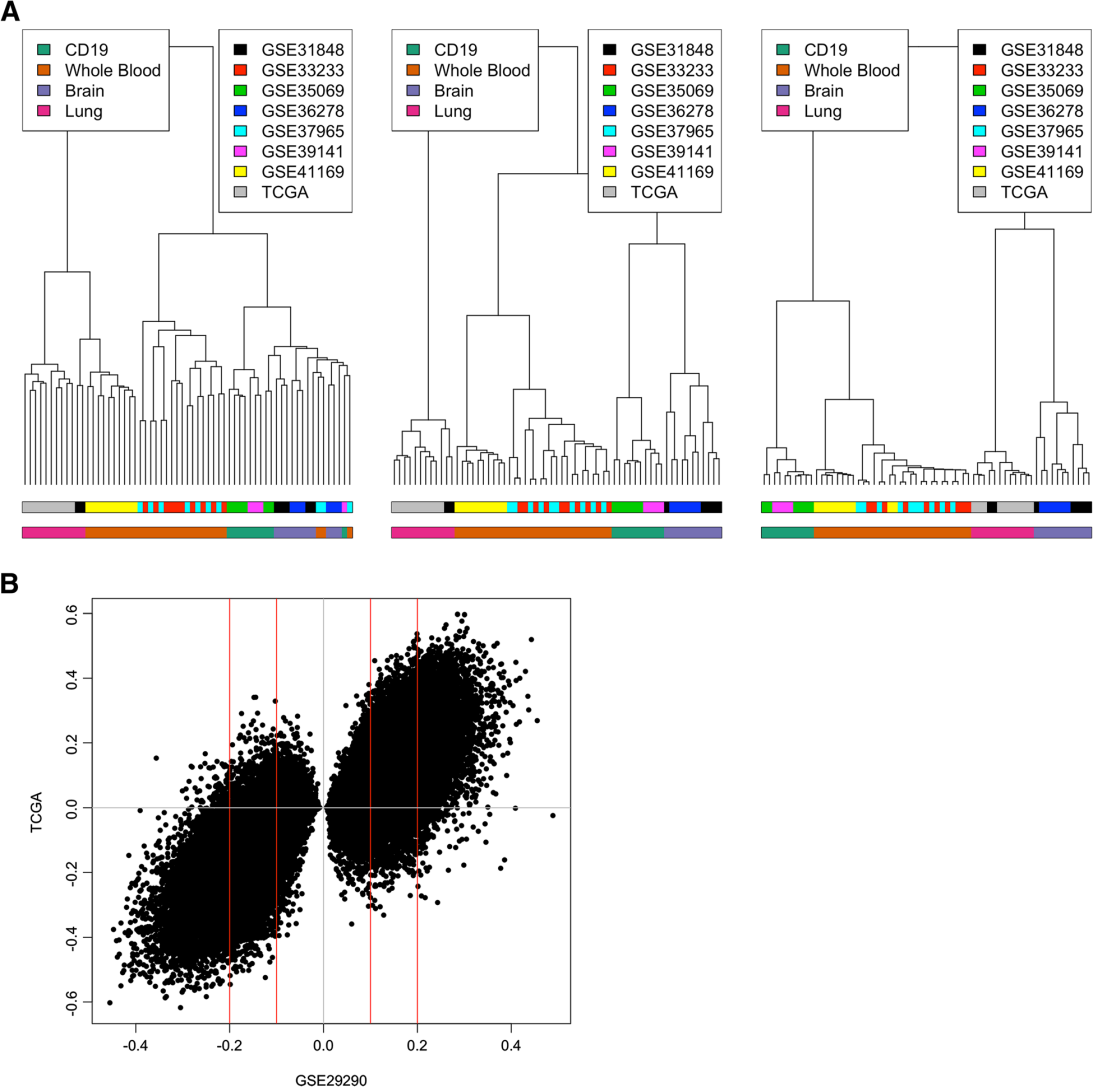
**Investigating batch effects. (A)** A plot of the hierarchical clustering of 4 different tissues (CD19, Whole Blood, Brain and Lung) from 8 different studies. A modified Euclidean distance was used so that only those probes with a 0-0.1 beta difference contributed (left panel), 0.1-0.2 beta difference (middle panel) or a 0.2-0.3 beta difference (right panel) contributed. **(B)** Breast cancer DMPs were called using an F-Test with corrected p-value < 0.01 for tumour samples against normal for a single dataset contained in Marmal-aid (GSE29290). The beta value difference for these DMPs was then plotted for another dataset of breast tumors against normal taken from TCGA. The red lines represent a threshold of an absolute beta value difference >0.1 and >0.2.

One cause for concern when combining data from a large number of experiments is the influence of batch effects. To investigate this further we selected a number of samples from 8 studies that included the same tissues from different experiments. We then modified the Euclidian distance calculation so that only absolute beta value differences of between 0 – 0.1 were used **(**Figure 3A Left Panel) to investigate the effect of small differences on the clustering. We found that the clustering was mostly based on experiment label rather than tissue type although this was not a perfect clustering. Similarly we calculated this modified Euclidian distance for absolute beta difference between 0.1 – 0.2 and 0.2 – 0.3. Both these larger differences showed clustering predominately based on tissue type although this was only perfect in the 0.2 – 0.3 beta difference range. Furthermore we chose to call differences (F-Test q-value < 0.01) between samples with breast cancer (8 samples) and those labelled as normal (8 samples) from GSE29290 and validate these calls in 8 randomly selected tumour samples and 8 randomly selected normal samples from TCGA (Figure 3B). We found that using no beta difference cut-off; 82% of the differences showed similar directionality in the TCGA data while using a >0.1 beta difference filter 92% showed similar directionality and finally using a >0.2 beta difference filter increased this further to 98%. Therefore it is important to be aware that when using publicly available databases interpreting small methylation differences can be dangerous.

## Conclusion

Marmal-aid is the largest publicly available Illumina Human 450 methylation database combining a number of public databases. While original raw data is available from Marmal-aid, we also provide processed data that has undergone normalisation as well as imputing missing data providing extra information not originally available. Marmal-aid also benefits from a standardised annotation format that has been hand annotated to allow for quick and easy searching and selection of available samples. Currently a large number of Illumina HumanMethylation450 experiments are submitted as beta values, which means there is a loss of information from the two channels in which the intensity is read. A number of normalisation techniques used for Illumina HumanMethylation450 data rely on this information and hence are not usable. It has been noted that a number of recent public submissions have included a RAW data file known as IDATs and in future we plan on including these into the database. We also plan on introducing future functions that will allow for initial QC of samples such as checks for contamination in blood samples or cell/tissue prediction algorithm as well as more automated meta-analysis of DMPs called in an experiment. Marmal-aid will be updated continually as more data becomes available and we aim to update at least once every two months.

## Availability and requirements

Project name: Marmal-aid

Project home page: http://marmal-aid.org

Operating system(s): Platform independent

Programming language: R

License: GPL-3

## Competing interests

The authors declare that they have no competing interests.

## Authors’ contributions

RL and VKR conceived the idea. RL developed the database. RL and VKR wrote the manuscript. Both authors read and approved the final manuscript.
